# A Rare Cause of Gastrointestinal Bleeding: Aorto-Enteric Fistula

**DOI:** 10.7759/cureus.27023

**Published:** 2022-07-19

**Authors:** Tejaswi Gadela, Mahati Paravathaneni, Deekshitha Manney, Harikrishna Bandla

**Affiliations:** 1 Internal Medicine, Bhaskar Medical College, Hyderabad, IND; 2 Internal Medicine, Mercy Catholic Medical Center, Philadelphia, USA; 3 Internal Medicine, Zaporizhzhia State Medical University, Zaporizhia, UKR; 4 Internal Medicine, Saint Francis Medical Center, Monroe, USA

**Keywords:** chiari’s triad, endovascular aortic repair, upper gi cancer, aorta-enteric fistula, massive gastrointestinal bleeding

## Abstract

Aorto-enteric fistula is defined as an abnormal connection between the gastrointestinal system and the aorta. The patients who develop this condition usually have a grim prognosis and the cases are universally fatal unless intervened with an endovascular repair or open surgical repair. Given the rarity and the relative unfamiliarity of this condition, an understanding of the presentation, pathogenesis, and management is vital to prevent catastrophic complications.

## Introduction

Aorto-enteric fistula is a catastrophic condition that develops when a part of the aortic wall erodes into the adjacent gastrointestinal tract. Although the exact incidence is difficult to estimate given the high case-fatality rate, it is estimated to be 0.007 per million [1]. Aorto-enteric fistulas may develop due to a multitude of predisposing conditions, including foreign body ingestion, aortic aneurysm, malignancy, previous history of gastric or aortic reconstruction surgery, peptic ulcer disease, or radiation. Rarely, infectious etiology, including syphilis, tuberculosis, and a bacterial or fungal aortic infection, may also lead to fistula formation between the aorta and the gastrointestinal tract [2-4]. The rarity of this condition frequently results in a low clinical suspicion and thereby causes fatal complications. Hence, timely intervention with endoscopic or open surgical repair is essential to prevent catastrophic complications.

## Case presentation

**Figure 1:**
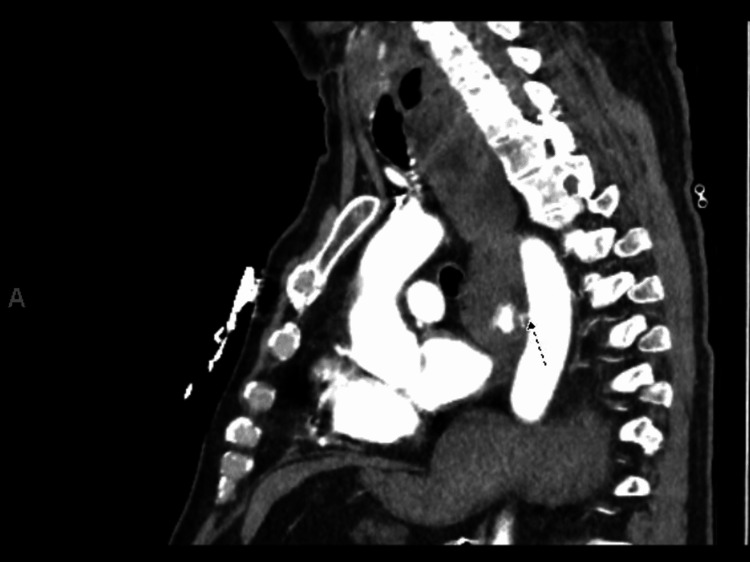
Sagittal view of computerized tomographic angiography scan showing active contrast extravasation into the lumen of esophagus or gastric cardia.

**Figure 2:**
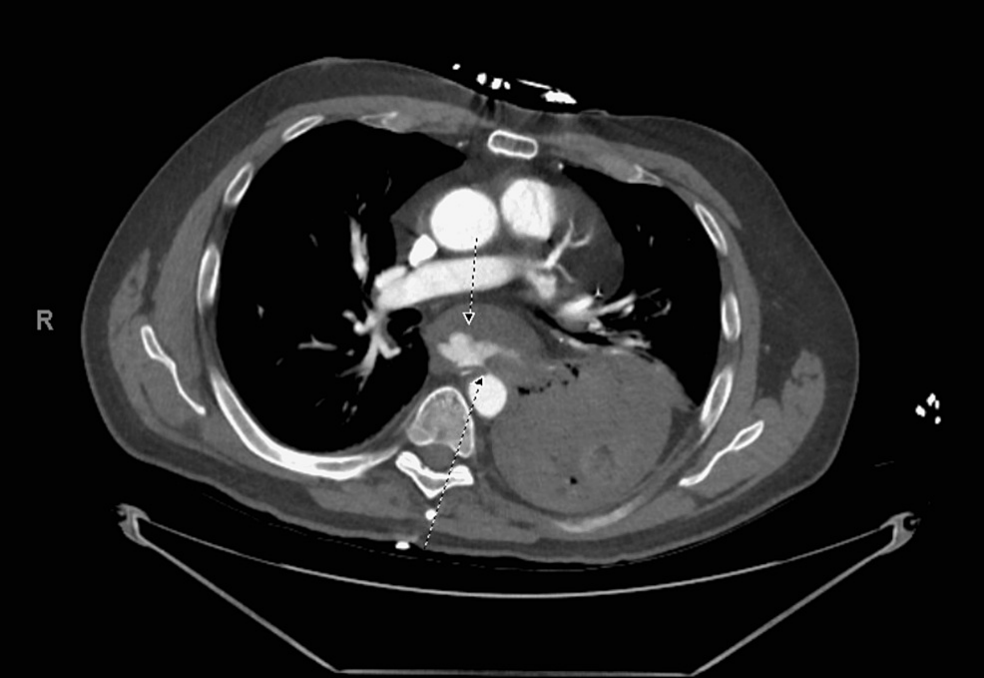
Axial view of computerized tomographic angiography scan showing active contrast extravasation into the lumen of esophagus or gastric cardia.

Our patient is a 60-year-old man who developed several episodes of bloody vomitus. He has a past medical history of esophageal adenocarcinoma treated with esophagectomy many years ago. The patient was in his usual state of health until a few hours prior to presentation when he developed a sudden onset of profuse, bloody vomitus without any symptoms of chest pain, shortness of breath, dizziness, or lightheadedness. He reported having about eight episodes prior to coming to the hospital. He denied any prior history of gastrointestinal bleeding, peptic ulcer disease, liver cirrhosis, or weight loss. However, he reported taking escalating doses of ibuprofen for new-onset lower back pain for the last few months. On presentation, the patient was noted to have a blood pressure of 110/70mm Hg and mild epigastric tenderness. Laboratory investigations were notable for hemoglobin of 14 g/dl, with no other significant abnormalities. The patient was admitted to the intensive care unit for closer monitoring, and gastroenterology was consulted for possible endoscopy for a high degree of suspicion for peptic ulcer disease. During this time, the patient had another episode of large volume hematemesis. He became profoundly hypotensive with a blood pressure of 78/50 mm Hg, necessitating an emergent computerized tomographic angiography scan of the abdomen and chest. Imaging revealed active contrast extravasation into the esophageal/gastric lumen demonstrating aorto-esophageal/gastric fistula to the descending thoracic aorta as well as a small hemothorax on the left side (Figures 1, 2).

Due to profuse hematemesis and concern for airway protection, the patient was intubated. The patient underwent an urgent endovascular repair of the fistula successfully. Postoperatively, after weaning the sedation, he developed right-sided weakness. Urgent computerized tomography (CT) scan of the head revealed a large middle cerebral artery vascular territory infarct involving the fronto-parieto-temporal lobes with a regional mass effect. The patient was not deemed to be a candidate for thrombectomy or tissue plasminogen activator. Repeat CT scan of the head showed a midline shift. Given a poor prognosis, the family decided to withdraw further care.

## Discussion

Aorto-enteric fistula is a devastating cause of upper gastrointestinal bleeding. Although the exact pathogenesis is unclear, animal studies have demonstrated the role of mechanical factors, especially the development of a pseudoaneurysm that predisposes to rupture and exsanguination into the gastrointestinal tract [5]. In addition to mechanical factors, some studies also suggest the role of inflammation within the aneurysmal wall that may cause proteolytic inflammatory cell degradation, causing pressure necrosis or mycotic erosion leading to fistula formation [6,7]. 

Although the presentation may vary depending on the etiology, patients usually present with gastrointestinal bleeding; the bleeding may range from a minor hemorrhage to massive, life-threatening hemorrhage or rapid overt exsanguination. The classic triad of symptoms is called the Chiari’s triad, which presents as pain, sentinel hemorrhage, and bleeding [8]. Typically, initial bleeding causes hypotension and clot formation causing the bleeding to temporarily stop. However, once the resuscitation efforts begin, the temporary clot dislodges and rebleeding occurs, resulting in massive exsanguination. The time between the initial bleeding and the exsanguination can range from hours to months, further emphasizing the need to maintain a high degree of suspicion [9].

The criticality of the clinical status frequently mandates emergency intervention before confirmation; however various diagnostic methodologies exist, including CT angiography, endoscopy, or arteriography to detect the abnormal communication [10-12]. Rapid diagnosis is essential as there is a limited time window before exsanguination ensues. However, it is important to recognize that these tools have limited sensitivity and specificity, and it is not unusual for the results to be negative or inconclusive. The CTA may be a valuable tool to evaluate the size and location of the aneurysm; however, active extravasation of contrast into the bowel may only be positive in 26% of the cases [13]. Although endoscopy is a primary diagnostic modality in a hemodynamically stable patient with upper GI bleeding, it rarely reveals the confirmatory evidence of aorto-enteric fistula as stable patients do not have active bleeding [13]. In an appropriate clinical setting, GI bleeding with unclear identifiable bleeding lesions may be considered a strong indicator for laparotomy [14,15]. 

Management of the aorto-enteric fistula entails hemodynamic and vascular support. The therapeutic approach involves open surgical intervention or endovascular repair. Although previous studies have shown no significant differences in overall mortality or postoperative complications with either option, endoscopic repair may have better short-term outcomes given lower intraoperative bleeding and decreased operative risk when compared to an open procedure [16]. Endovascular repair may also be considered in patients who are not candidates for emergency surgery or as a bridge to conventional surgery for a few hours or days to achieve short-term hemostasis and optimization [17]. Without treatment, aorto-enteric fistula is frequently fatal, with a significantly high mortality rate of 80-100% [18-20]. 

## Conclusions

Despite technological and diagnostic advancements, aorto-enteric fistula remains a life-threatening condition with a high degree of morbidity and mortality. Varied clinical presentation, overlapping features with other causes of GI bleeding, and frequent insidious episodes of gastrointestinal bleeding that are frequently undiagnosed until massive exsanguination makes the diagnosis challenging. Hence, a focused clinical approach and multidisciplinary management are essential to diagnose and manage this catastrophic condition appropriately. Physicians must also maintain a high degree of clinical suspicion, especially in patients with aortic aneurysms or prior gastric or aortic reconstruction surgeries.
